# Comparing the Effects of Aerobic and Stretching Exercises on the Intensity of Primary Dysmenorrhea in the Students of Universities of Bushehr

**Published:** 2015-03

**Authors:** Farideh Vaziri, Azam Hoseini, Farahnaz Kamali, Khadijeh Abdali, Mohamadjavad Hadianfard, Mehrab Sayadi

**Affiliations:** 1 Department of midwifery, School of Nursing and Midwifery, Shiraz University of Medical Sciences, Shiraz, Iran; 2 Department of midwifery, School of Nursing and Midwifery, Bushehr University of Medical Sciences, Bushehr, Iran; 3 Department of Physical Medicine and Rehabilitation, Medical School, Shiraz University of Medical Sciences, Shiraz, Iran; 4 Health Policy Research Center, Shiraz University of Medical Sciences, Shiraz, Iran

**Keywords:** Primary dysmenorrhea, Aerobic exercise, Stretching exercise, Exercise

## Abstract

**Objective:** To compare the effects of aerobic and stretching exercises on severity of primary dysmenorrhea.

**Materials and methods:** This randomized clinical trial was conducted on 105 female students who were suffering from primary dysmenorrhea. The participants were divided into aerobic exercise, stretching exercise, and control groups. The two intervention groups did the exercises three times a week for eight weeks (two menstrual cycles). The intensity of dysmenorrhea was determined using a modified questionnaire that assessed several symptoms of dysmenorrhea. After all, the data were compared between and within groups through analysis of variance.

**Results:** Before the intervention, the mean intensity of dysmenorrhea was 40.38 ± 5.5, 37.40 ± 3.8, and 38.45±3.3 in aerobic, stretching, and control groups, respectively, but the difference was not statistically significant. After the intervention, however, a significant difference was found among the three groups regarding the mean intensity of dysmenorrhea in the first and second menstrual cycles. Also, a significant difference was observed between the aerobic group and the control group as well as between the stretching group and the control group. Within group comparisons showed a significant difference in the aerobic and the stretching group before and after the interventions. However, no such difference was observed in control group.

**Conclusion:** Both aerobic and stretching exercises were effective in reducing the severity of dysmenorrhea. Therefore, women could choose one of these two methods with regard to their interest and lifestyle.

## Introduction

Over 50% of the women in the reproductive ages suffer from painful menstruation; among them, 10% have severe dysmenorrhea and 1 to 3 days of their life is impaired each month (1). Primary dysmenorrhea starts some hours before menstruation and continues up to 12-72 hours and is like delivery of pains along with cramps in the lower abdomen radiating toward the inner side of the thighs. Half of such cases experience systemic symptoms, such as nausea, vomiting, diarrhea, fatigue, irritability, and dizziness (2, 3). Even though the causes of primary dysmenorrhea are still not clearly determined, but it has been demonstrated that prostaglandin plays a major role in its occurrence, and most of its symptoms could be justified by prostaglandin activity (2, 4).

During the recent 20-30 years, regular exercise and physical activities have been introduced as effective methods for prevention and treatment of dysmenorrhea (3, 5, 6). Exercising affects the levels of steroid hormones in blood circulation of the women in reproductive ages (7, 8). Moreover, the elevation of the endorphin hormone leads to an increase in pain threshold (4, 5, 9).

Since stress can increase the activity of the sympathetic system leading to increased uterine muscle contraction, it can increase the symptoms of pre-menstruation syndrome (PMS). Exercise can thus reduce the activity of the sympathetic system, resulting in a decrease of dysmenorrhea symptoms (3).

Although it appears that doing exercise can relieve the pain associated with dysmenorrhea, some observational studies in this area have provided controversial results. Some researchers have reported that exercise can improve dysmenorrhea, while some others have found that regular physical activities can worsen the symptoms of dysmenorrhea (2, 3). In an observational study that Hightower conducted on 21 sedentary and 20 healthy active women, active participants were found to have lower intensity of dysmenorrhea (10). Similarly, Dyusk performed a study on 67 athletic women and 96 non-athletic high school girls and found that the intensity of dysmenorrhea was significantly lower in the athletic group (11). Inversely, Jart et al. indicated no significant difference between the women who practiced aerobic exercises and others in terms of dysmenorrhea (12). Also, Metheny and Smith conducted a study on 179 nursing students and revealed that the participants with regular exercise had significantly more severe dysmenorrhea compared to those who did not exercise (13).

One randomized experimental study which was conducted on 36 participants over 25 years ago revealed the effect of aerobic exercises on primary dysmenorrhea (14). In a review study, Daley concluded that doing exercise had no clear effects on primary dysmenorrhea. However, considering the positive effects of exercise on health, the effects of exercise on dysmenorrhea can be discussed (15). Nevertheless, recent studies on female students in Iran have demonstrated the positive effects of exercise on primary dysmenorrhea (16, 17, 18).

Although few clinical studies have been conducted on the effects of exercising on primary dysmenorrhea, but no studies have compared the effects of the two mentioned exercises on dysmenorrhea. Thus, the present study aims to compare the effects of stretching and aerobic exercises on prevention and treatment of dysmenorrhea.

## Materials and methods

The research population consisted of all the female students of the universities of Bushehr in 2012-2013. In this study, the participants were divided into two intervention groups (stretching and aerobic exercises) and a control group. Based on the research objectives and the previous studies conducted on the same issue, considering α = 0.05, power of 80%, and variance of 1.5, and using the following formula, a 105-subject sample size (35 in each group) was determined for the study:

The participants were selected through purposive and convenience sampling and were divided into three groups using permuted-block randomization. Then, the research objectives were explained to the participants and their written informed consents were obtained.

The inclusion criteria of the study were being single, being between 18 and 30 years old, having no history of mental and physical diseases, having no history of joint, motion, muscle, and bone diseases that reduce their abilities to exercise, not being professional athletes, not having taken any medications or vitamin and mineral supplements during three menstrual cycles before the trial, not having done leisure time exercises leading to sweating or increasing heart rate (using Godin's questionnaire) in the recent three months, not passing their physical education course, not suffering from pelvic diseases such as endometriosis, myoma, and ovarian cyst, Body Mass Index( BMI) of 19-29, and having regular menstrual cycles of 24-35 days.

On the other hand, the exclusion criteria of the study were being absent for more than two sessions of exercise in both menstrual cycles, beginning of doing any type of exercise in the control group, beginning of any additional exercises in the intervention groups, not being able to tolerate physical exercises, and occurrence of any unexpected events, such as death of relatives or marriage.

The intensity of dysmenorrhea was determined using a modified questionnaire containing 14 questions adopted from Menstrual Symptom Questionnaire (MSQ) (19). In this questionnaire, the intensity of the symptoms was scored as 1 to 5 representing no symptoms and very severe symptoms, respectively, (table4). The individuals who had the menstruation cramps of at least 3-5 (moderate to very severe) were entered into the study. This questionnaire was completed by the participants in all the three groups for three times; before the intervention, at the end of the first cycle of the intervention, and at the end of the second cycle. The reliability of this questionnaire was measured using test-retest method and Pearson's correlation coefficient (0.88). 

In the aerobic exercise group, aerobic exercises were performed on a treadmill device for 20 min (four 5-min stages). The exercises started with low intensity and their intensity was increased in the second and third stages. In the fourth stage, the intensity of the exercises was reduced again; thus, the intensity of exercises was the same in the first and fourth stages. It should be mentioned that the intensity of exercises was equal for all the participants in this group. Each participant performed these exercises three times a week for two menstrual cycles. Using the treadmill was taught to the participants according to the protocol. The participants did the exercises in the sports club of Bushehr University of Medical Sciences.

In the stretching exercise group, the participants had to do 10 stretching exercises in abdomen, pelvis, and groin that were performed 3 days a week for two menstrual cycles (17). Each exercise started with 10 second in the first session and 1 second was added to it every session. Besides, each movement was repeated for 5 times. Stretching exercises were taught to the participants by a trainer who also supervised their exercises in the sports club of Bushehr University of Medical Sciences. Figures of these exercises are available in the appendix. 

The control group did not do any exercises and did not take any chemical or herbal drugs or supplements to prevent their dysmenorrhea during their menstrual cycles. After data collection, the data were entered into the SPSS statistical software (v. 16) and analyzed using descriptive statistics, ANOVA, repeated-measures ANOVA, and post-hoc test. Besides, P < 0.05 was considered as statistically significant.

## Results

In this study, 176 female students studying in universities of Bushehr were interviewed, among whom 23 students refused to participate and 53 were excluded due to lack of the inclusion criteria. After randomization, 3 and 4 participants were excluded in the first and second intervention cycles, respectively, due to lack of cooperation. Finally, the data of 98 participants were statistically analyzed.

The mean age of the participants was 20.77 ± 0.197 years and their mean BMI was 24.44 ± 2.82. In addition, most of the participants were second year students and second child of their family. The results revealed no statistically significant difference between the study groups in terms of baseline characteristics (Table 1). 

The duration of dysmenorrhea was 6-12 hours, up to 24 hours, and more than 24 hours in 38 (38.8%), 43 (43.9%), and 17 participants (17.3%), respectively. Moreover, 63 participants (65.3%) used analgesics, 13 ones (13.3%) rested, (11 ones (11.2%) used topical heating, 10 ones (10.2%) used herbal remedies, and 1 participant (0.1%) used no methods for relieving dysmenorrhea. Furthermore, 53.1% of the participants (52 ones) described dysmenorrhea as a debilitating event, 37.8% (37 ones) described it as a disturbing event, and 9.2% (9 ones) considered it as a normal event. Other information related to menstrual characteristics is presented in Table 1.

The total mean score of the dysmenorrhea intensity was calculated for the three groups in the baseline as well as in the first and second cycles of the intervention. The study results indicated no significant difference among the three groups regarding the intensity of dysmenorrhea before the intervention. However, a significant difference was observed among the three groups in this regard in the first and second cycles of the intervention (Table 2). Also, intra-group comparisons showed a significant difference in the mean intensity of dysmenorrhea in the baseline, first, and second cycles in the two intervention groups (P < 0.01), but not in the control group (P = 0.220).

The three groups were compared two-by-two using post-hoc test. The results showed a significant difference between the two intervention groups and the control group concerning the mean intensity of dysmenorrhea in the first and second cycles. However, no significant difference was observed between the aerobic and stretching exercise groups. (Table 3).

Evaluation of the mean intensity of each symptom of dysmenorrhea in the whole sample before the intervention demonstrated that the lowest mean score was related to constipation during menstruation (1.4 ± 0.79), while the highest one was related to backache at the beginning of menstruation (4.05 ± 0.466).

## Discussion

The results showed that each of these two types of exercises could reduce the intensity of dysmenorrhea compared to the control group. However, none of these two types of exercises was superior to the other in terms of reducing the intensity of dysmenorrhea.

In the literature review, no studies were found which compare the effects of these two exercises on the intensity of dysmenorrhea and it seems that this work was the first one in this regard. 

Jahromi et al. studied the effects of a period of weight training exercises on the features of menstrual cycle in 250 students of Shiraz University in a single group before and after doing the exercises. The results showed that the intensity of dysmenorrhea significantly decreased after doing the exercises compared to the beginning of the study (8). Additionally, Anour et al. examined the effect of simple exercises that can be done at home, on dysmenorrhea and life quality of 40 women. The intervention consisted of 10 min stretching exercises, 20 min aerobic exercises such as walking and cycling, and 10 min relaxation every day, which was continued for three months. The participants' intensity of pain was measured using Visual Analogue Scale (VAS).

**Table 1:** Comparison of the baseline characteristics in the three study groups

	**Age**	**BMI** *	**Number of child**	**Year of education**	**Menarch age**	**Duration of menstruation**	**interval of mensturation**
	Mean ± SD**	Mean ± SD	MED (IQR)***	MED (IQR)	Mean ± SD	Mean ± SD	Mean ± SD
**Control**	**1.83** **±** **20.43**	**4.21** **±** **24.07**	**2(1-3)**	**2(1-3)**	**68** **.** **0 ** **±** ** 13.21**	**1.37** **±** **6.18**	**2.28** **±** **29.24**
**Stretching**	**1.94** **±** **20.81**	**1.72** **±** **24.58**	**2(1-3)**	**2(2-3)**	**66** **.** **0 ** **±** ** 12.35**	**1.19** **±** **24. 6**	**2.34** **±** **28.39**
**Aerobic**	**2.07 ** **±21.10**	**1.48 ** **±24.72**	**2(1-3)**	**2(2-3)**	**58** **.** **0** **±** **13.27**	**24** **.** **1 ** **±6.11**	**2.58** **±** **30.41**
**Total **	**1.97** **±** **20.77**	**2.82** ** ±** **24.44**	**2(1-3)**	**2(1-3)**	**0.64 ** **±** ** 12.94**	**1.26 ** **±6.17**	**2.41 ** **±29.34**
**p-value**	**0.378**	**0.613**	**0.959**	**0.374**	**0.454**	**0.435**	**0.373**

* Body Mass Index;

** Standard deviation;

*** Median (interquartile range)

**Table 2:** Comparison of the intensity of dysmenorrhea in the three groups (Mean ± SD)

	Before intervention	First cycle of intervention	Second cycle of intervention	P value
**Control**	**38.45 ± 3.3**	**38.11 ± 3.6**	**36.97 ± 4.3**	**0.220**
**Stretching**	**37.40 ± 3.8**	**37.40 ± 4.6**	**23.21 ± 6.8**	**0.000**
**Aerobic**	**40.38 ± 5.5**	**32.48 ± 5.8**	**23.21 ± 6.8**	**0.000**
**P value**	**0.380**	**0.000**	**0.000**	

**Table 3:** Paired comparison of the study groups regarding the intensity of dysmenorrhea (Mean ± SD)

	Control	Stretching	p-value	Control	Aerobic	p-value	Stretching	Aerobic	p-value
**Before intervention**	**38.45 ± 3.3**	**37.40 ± 3.8**	**0.969**	**38.45 ± 3.3**	**40.38 ± 5.5**	**0.220**	**37.40 ± 3.8**	**40.38 ± 5.5**	**0.022**
**First cycle of intervention**	**38.11 ± 3.6**	**37.40 ± 4.6**	**0.000**	**38.11 ± 3.6**	**32.48 ± 5.8**	**0.000**	**37.40 ± 4.6**	**32.48 ± 5.8**	**0.064**
**Second cycle of intervention**	**36.97 ± 4.3**	**23.21 ± 6.8**	**0.000**	**36.97 ± 4.3**	**25.38 ± 7.5**	**0.000**	**23.21 ± 6.8**	**25.38 ± 7.5**	**0.529**

The results demonstrated that, the intensity of dysmenorrhea decreased, which continued in the following two months, as well (20). Also, several recent studies have confirmed the positive effects of stretching exercises on the intensity of dysmenorrhea in high school and university student girls (17, 9, 21, 22).

Medical literature indicates that doing exercises is effective in women's other complaints about menstrual cycle, such as PMS (23, 24). 

In contrast to the above-mentioned studies, Blakey et al. examined 594 students using a questionnaire and found no relationships between doing exercises and dysmenorrhea (25). Meta-analysis of observational studies conducted by Latthe et al. also showed that exercises could slightly reduce the risk of dysmenorrhea (26).

In the present study, prostaglandins which have a crucial role in creating the symptoms of dysmenorrhea were not measured before and after the intervention, and no studies were also found on this issue. On the other hand, the intensity of dysmenorrhea has been shown to be higher in the women with more stress, and doing exercises has been found to be effective in the reduction of stress (23, 27, 28).

Menstrual pain may be resulted from increased contraction of uterine muscle which is innervated by the sympathetic nervous system. Stress is supposed to increase the sympathetic activity which may lead to the increase of menstrual pain by enhancing the intensity of uterine contraction. So, due to the fact that exercise could reduce and moderate stress, the sympathetic activity may be decreased. Thereby, intensity of menstrual pain and other related symptoms may be reduced as well. Another possible dilemma in this respect is that, since performing physical activity leads to the release of endorphins which are produced by brain, the pain threshold could be enhanced (17, 29).

The present study used a modified questionnaire which categorized the symptoms of dysmenorrhea and measured their intensity. However, most of the above-mentioned studies have considered dysmenorrhea as uterine cramps and used VAS for measurement of pain. The present work showed that exercising had positive effects on all menstrual symptoms.

Future studies are recommended to evaluate the participants' psychological variables and social stresses, so that the results will be more indicative of the exercising effects on the intensity of primary dysmenorrhea.

## Conclusion

The findings of the current study showed that both types of aerobic and stretching exercises were effective in reducing the intensity of dysmenorrhea. Given the similar effects of these two types of exercises on primary dysmenorrhea, women can choose either of them depending on their conditions and interest.
